# Hypovitaminosis D in Young Basketball Players: Association with Jumping and Hopping Performance Considering Gender

**DOI:** 10.3390/ijerph18105446

**Published:** 2021-05-19

**Authors:** Borja Ricart, Pablo Monteagudo, Cristina Blasco-Lafarga

**Affiliations:** 1 Sport Performance and Physical Fitness Research Group (UIRFIDE), Physical Education and Sports Department, University of Valencia, 46010 Valencia, Spain; M.Cristina.Blasco@uv.es; 2 Alqueria LAB, Valencia Basket, 46013 Valencia, Spain; 3 Education and Specific Didactics Department, Jaume I University, 12071 Castellón, Spain

**Keywords:** vitamin D, explosive strength, performance, nutrition, training

## Abstract

This study aimed to verify whether a group of young well-trained basketball players presented deficiencies in vitamin D concentration, and to analyze whether there was an association between vitamin D concentration and jumping and hopping performance. Gender differences were considered. Twenty-seven players from an international high-level basketball club (14 female, 16.00 ± 0.55 years; 13 male, 15.54 ± 0.52 years) participated in this cross-sectional study. Rate of force development was evaluated by means of the Abalakov test (bilateral: AbB; right leg: AbR; left leg: AbL); and the triple hop test (right leg: THR; left leg: THL). Blood samples were collected for the determination of serum 25-hydroxyvitamin D and nutritional status. Vitamin D insufficiency was found in both women (29.14 ± 6.08 ng/mL) and men (28.92 ± 6.40 ng/mL), with no gender differences regarding nutritional scores. Jumping and hopping performance was confirmed to be significantly larger in males (AbL, THR, and THL *p* < 0.005), whose CV% were always smaller. A positive correlation was found between AbB and vitamin D (*r* = 0.703) in males, whereas this correlation was negative (−0.611) for females, who also presented a negative correlation (*r* = −0.666) between THR and vitamin D. A prevalence of hypovitaminosis D was confirmed in young elite athletes training indoors. Nutritional (i.e., calciferol) controls should be conducted throughout the season. Furthermore, whilst performance seems to be affected by low levels of this vitamin in men, these deficiencies appear to have a different association with jumping and hopping in women, pointing to different performance mechanisms. Further studies accounting for differences in training and other factors might delve into these gender differences.

## 1. Introduction

Nutrition plays an important role in the health and performance of athletes. In particular, vitamins are essential in various processes, including hemoglobin synthesis, maintenance of bone health, immune function, protection against oxidative damage, neuronal functions, and the synthesis and repair of muscle tissue during recovery from injury [1,2]. Over the last decade, the monitoring of vitamin D, or calciferol, a fat-soluble vitamin with the structure of a steroid hormone that is functionally different from all others, has been of particular interest [3]. We refer to vitamin D3, a vital isomer synthesized in the cell membrane of the epidermis and dermis as a response to solar radiation, as its other common form, D2, is derived from plants and is impossible for the human body to synthesize [4,5].

Vitamin D3 regulates the expression of more than 900 gene variants, which in turn significantly [6] impacts numerous functions related to sporting performance. Among other things, it is involved in the regulation of exercise-induced inflammation, neurological function, cardiovascular health, glucose metabolism, bone health, and skeletal muscle performance [7]. More specifically, it is attributed with an ergogenic effect on neuromuscular efficiency and the muscle-contraction mechanism [8,9], as well as optimizing acute adaptive response to physical exercise [10], so that performance in athletes may be affected by deficient levels of this vitamin [11,12].

However, recent research suggests that high-performance athletes are at constant risk of vitamin D deficiency, increasing the risk of stress fractures, acute illness, and sub-optimal muscle function [3]. In addition to a possible nutritional deficit due to insufficient calorie intake in athletes with high energy needs [13], or poor diet [14], vitamin D deficiency has been linked to a lack of or drastic reduction in vitamin D production in the winter months due to a lower incidence of sun on the skin [15]. For example, Bescos and Rodriguez [16] found that more than half of one professional basketball team had hypovitaminosis D after the winter. More recently, Fishman et al. [17] found a high prevalence of vitamin D insufficiency in National Basketball Association (NBA) players.

Therefore, it seems that vitamin D deficiency is accentuated in athletes who train and compete indoors throughout the year, as is the case of basketball. Taking also into account the relationship between vitamin D and the aforementioned optimization of muscle contraction [8,9] and/or prevention of bone health issues [7], it seems that this deficiency is particularly important in a sport that involves continuous accelerations and braking, jumps and receptions [18]. The rate of force development in the lower extremity is of the utmost importance [19,20], and the risk of musculoskeletal injuries is high [21]. Moreover, jumping, which may be affected by calciferol deficit, is one of the most common actions performed in this sport [22,23], with between 40 and 60 jumps being made per athlete during a single game [24]. Jumping is also one of the most common ways of assessing player performance [25], condition-maturity level [26,27], level of functional health over the course of the season [28,29], and sporting life success [30,31].

Knowing whether basketball players are calciferol deficient from their early formative stages, and the possible relationship between their vitamin concentrations and muscle function as assessed by jumping, is therefore of interest to the medical and technical staff who care for these athletes. Although there is no evidence to suggest gender differences in vitamin D intake [32] and/or deficit [33,34], differences between male and female basketball players tend to be significant in jumping ability [35], so it is equally important to analyze these associations while taking gender into account. The aims of this study are, therefore, to test whether a group of young high-performance basketball players are vitamin D deficient (1); and to analyze whether there is any relationship between vitamin D levels and muscle strength performance as measured by two types of jumps (2), taking into account gender differences. To our knowledge, no studies have previously investigated this potential relationship.

## 2. Materials and Methods

### 2.1. Participants

This quantitative, descriptive, and correlational study involved 27 young basketball players belonging to a top-level competitive club in the ACB (Asociación de Clubes de Baloncesto) league, of whom 14 were girls (16.00 ± 0.55 years, all of them had attained menarche), and 13 were boys (15.54 ± 0.52 years). Before data collection began, both the subjects and their legal guardians were informed of the purpose of the study. Each participant signed an informed consent form, agreeing to participate in the study, which had been approved by the ethics committee of the local university (H1553774899546).

### 2.2. Protocol

The data collection was carried out during the regular season in the month of December, and on three alternate days of the same week. The week prior to the first assessment, the participants were informed that they should consume no stimulant drinks (caffeine or energy drinks); they could not eat two hours prior to the tests; and they should maintain their normal nutritional habits. The first evaluation session involved blood tests. In the second session, the anthropometric measurements of the players were taken and the Abalakov vertical jump test was performed first bilaterally (both legs at a time), and then unilaterally (one leg at a time). In the final evaluation session, data on the triple hop test were collected. Prior to the jumping tests, a standardized 10-min warm-up was performed on both days, consisting of jogging, dynamic stretching, lower and upper limb strength exercises, plyometric exercises, and high-intensity running with changes of direction. No familiarization phase was carried out for the evaluation tests, as all of the athletes had already taken these at some point during the season.

### 2.3. Assessment Tools

#### 2.3.1. Blood Test

The method for determining the body’s vitamin D status consisted of measuring the serum 25-hydroxyvitamin D concentrations [36]. For many years, there has been a consensus that blood concentrations of this metabolite reflect total body vitamin D, including endogenous synthesis by exposure to sunlight, dietary intake in supplemented or unsupplemented meals, and drug treatments [37]. The blood samples were taken by a medical professional from a hospital in the same city. The players were summoned to the medical center, along with their fathers, mothers, or legal guardians, with an overnight fast required before attending.

For the blood tests, 5 mL of venous blood were extracted from the antecubital vein of each participant. Once obtained, the blood samples were allowed to clot and then centrifuged at 3000 rpm for 10 min at room temperature to isolate the serum. The serum was aliquoted into an Eppendorf tube and conserved at −80 °C until biochemical analysis. Serum vitamin D concentrations were determined using the LIAISON 25(OH) Vitamin D TOTAL Assay (CLIA) (Eurofins Megalab S.A., Valencia, Spain), which is a direct competitive chemiluminescence immunoassay for human serum intended for use on the DiaSorin LIAISON automated analyzer (DiaSorin S.P.A., Saluggia, Italy). Once the laboratory tests had been performed, the reports containing the analytical data were submitted to the researchers for further analysis.

#### 2.3.2. Anthropometric Measurements

Mass (kg) and height (cm) measurements were recorded using a scale (SECA 769, CE 0123, Hamburg, Germany) and a stadiometer (SECA 220, CE 0123, Hamburg, Germany). The body mass index (BMI) of the participants was calculated using the formula mass/height^2^ (kg/cm^2^).

#### 2.3.3. Abalakov Test

In order to evaluate the rate of force development of the lower extremity, the Abalakov test [38] was performed both bilaterally (Ab) and unilaterally (Abalakov right or AbR; and Abalakov left or AbL), with the height of the jump being recorded. All players performed three jumps in each modality, with a recovery period of two minutes between the jumps [39], although only the best jump in each modality was included in the statistical analysis. The jumps were recorded using a Din-A2 contact platform (420 × 594 mm) and Chronojump software (Boscosystem^®^, Barcelona, Spain).

#### 2.3.4. Triple Hop Test

To evaluate the power and neuromuscular control of a horizontal jump, the participants took the triple hop test [40,41]. This test consists of three consecutive jumps on one leg, with the distance reached after the last jump being recorded [40]. Each player performed the test twice with each leg alternatively (triple hop left or THL; and triple hop right or THR), and the best jump with each leg was used in the subsequent analysis. A standard 12-metre tape measure was used to measure each jump.

### 2.4. Statistical Analysis

The data were analyzed using the statistics package SPSS v23 for Windows (SPSS Inc. Chicago, IL, USA). Once the normality of the sample had been analyzed (Shapiro–Wilk test), the descriptive variables were then calculated and expressed as the mean and standard deviation (mean ± *SD*). *T*-tests for independent samples or Mann Whitney U-tests were performed to analyze whether there were sex-related differences between the main study variables. *T*-tests for related samples and the Wilcoxon test were also performed to compare whether there were sex-related asymmetries between the legs. To check whether there was a relationship between vitamin D levels and performances in the jumping tests, we performed a correlation analysis (Pearson’s *R* or Spearman’s Rho according to the normality), both with and without controlling for the covariate BMI. Statistical significance was set at *p* < 0.05, with the absolute correlation coefficients considered being: *r* < 0.1, trivial; 0.1–0.3, low; 0.3–0.5, moderate; 0.5–0.7, strong; 0.7–0.9, very strong; >0.9, almost perfect; and 1, perfect [42].

## 3. Results

The final sample comprised 14 girls (16.00 ± 0.55 years, 174.20 ± 6.35 cm, 67.98 ± 6.73 kg) and 13 boys (15.54 ± 0.52 years, 190.73 ± 6.45 cm, 78.17 ± 8.87 kg). No significant differences were found between boys and girls in terms of age, but significant differences were found for weight and height (*p* < 0.05), with higher values recorded in the boys. Table 1 presents the results of the main blood composition parameters. No significant differences between boys and girls were found for any of the items, and the coefficients of variation were generally high in both cases.

Table 2 shows the values obtained in the neuromuscular performance tests, with lower coefficients of variation with respect to the analytical assessment, and even greater homogeneity among the boys. When analyzing the differences by sex, significant differences (*p* < 0.01) were observed in the Abalakov test for the left leg. Significant differences were also found in the triple hop test, both for the left leg (*p* < 0.001) and right leg (*p* < 0.001). Finally, significant differences were found in boys (*p* < 0.010) between the results for the right and left legs in the Abalakov test.

Table 3 shows the correlation analyses between vitamin D concentration and the results of the neuromuscular performance tests. While in boys, a high positive correlation was found between the Abalakov test (performed in a bipedal manner) and serum vitamin D concentration, in girls this relationship was also high, but negative. When BMI was considered as a covariate, the correlation coefficient increased slightly in boys, while it decreased in girls. There was also a high negative correlation between the triple hop test performed with the right leg and vitamin D in girls, which in this case increased slightly when considering BMI.

## 4. Discussion

For the first objective of this study (to check whether young basketball players of a formative age suffer from vitamin D deficiency), our results confirm that both girls and boys show this deficiency at the age of 14–16, while the other components analyzed were found to be within the normal range. As for whether this deficit could influence explosive strength as assessed by jumping, the second objective of this study, the data reveals that at these ages there is no association between these variables when considering the sample as a whole. However, when taking sex into account, the data points to differences regarding the correlations in young players of the two sexes, while at the same time the expected differences are observed in the rate of force development in some of the jumps that are determining factors for basketball performance (AbL, THR, and THL).

According to the levels previously established by some authors [43], young players of both sexes already suffer vitamin D insufficiency (20–30 ng/mL), while they present normal values for the other blood components [44,45,46,47,48]. Our results are, therefore, consistent with other studies that have shown low concentrations of vitamin D in elite athletes [49,50], with up to 56% of one sample of athletes being below the levels considered adequate [51]. In agreement with other studies [33,34] there were no differences between sexes in the vitamin D deficiencies.

As previously noted, indoor sports involve a vitamin D deficiency rate almost twice that of outdoor sports [52]. Seasonal variation in the levels of this vitamin has also been observed [15,53]. This seasonal variation should be taken into account, as it has been observed that athletes who are vitamin D deficient during the winter are at a higher risk of having lower levels in the spring [54]. This latter period is one of the most important phases of the season since the final rankings are decided and, moreover, there are more matches, therefore leading to a greater risk of fatigue and injury [55]. Both indoor training and seasonal variation are associated with low sun and ultraviolet (UVB) exposure, the main source from which the body synthesizes this vitamin [56]. It seems important, therefore, to monitor 25(OH)D concentrations throughout the basketball season in order to mitigate any potential effects that this insufficiency may cause for the players, despite the fact that these are initial stages in which they are still training and competing quite below the level of professional athletes [57,58].

Based on this, the second objective of this study was to find out whether lower vitamin D concentrations could influence basketball performance (by assessing the rate of force development of the players through two different types of jumps). Although we did not find sex differences regarding vitamin D, all the analyses were also performed considering the sex of the participants because individual differences in jumping ability in male and female basketball players tend to be significant [35]. Our data reinforces the importance of always considering these sex differences when analyzing performance, because although there is no association between these variables when considering the entire sample in general, the data does reveal different results for men and women.

On the one hand, there is a very strong positive correlation seen in the boys between vitamin D and the bilateral Abalakov test, with a correlation coefficient that increases even more when BMI is considered as a covariate. Some authors have argued that this vitamin increases the size and number of type II muscle fibers [54,59], which could influence an athlete’s jumping ability. However, this association is negative in the case of the girls, and decreases when BMI is taken into account. These results differ from those obtained by Ward et al. [60], who concluded that vitamin D was significantly associated with muscle strength in adolescent girls, although the participants in that study were not athletes.

In this sense, it is important to emphasize that at this age, boys may be less mature than their female peers [61]. Even close to full maturity, less vitamin D does not imply less jumping ability in these young female players, but rather the opposite, suggesting that there may be other mechanisms (for example, those related to good intermuscular coordination) that help these girls to jump more. Not surprisingly, the jumps where sex-related differences were found (AbL, THL, and THR), presented the lowest coefficient of variation in the boys, with these being clearly lower for the boys than their female counterparts for these same jumps. Further studies involving larger sample sizes and a more heterogeneous performance profile for girls should confirm whether, as it appears, only their male counterparts are likely to rely more heavily on explosive force production rates, with vitamin D concentration exerting a positive influence on this variable.

Considering the previous reasoning, the game and specific training would not have highlighted differences between the right and left leg in girls when performing the Abalakov test in a unilateral manner, again contrary to that seen in boys (with a significantly better AbL than AbR, and, indeed, higher AbR and AbL than those of the more mature girls in this study). As pointed out by Jones and Bampouras [62], the dominant leg of male athletes tends to present higher strength values than the non-dominant leg, which could explain the difference recorded for our male athletes. The reason behind why we found no association between vitamin D and the unilateral tests in men could be related to a lack of stability during these movements due to coordination problems [63]; to perform well in unilateral tests, an individual must have adequate balance, coordination, muscle strength, and neuromuscular control [64], and not just rate of force development. This would account for why we only found the correlation in the bilateral test, where it is easier to coordinate movements and thereby apply a greater amount of force.

The fact that the girls did not show significant asymmetries between legs suggests that women do not tend to have a more dominant leg [65]. This information, together with the fact that the strength values (performance in cm) produced by the trainee players in our study are already similar to those obtained by professional athletes [66], could indicate that the potential for further improving this ability in women may be limited, and that jumping and hopping ability may not be the most determining factor in terms of becoming a professional player. This suggests that adequate levels of vitamin D are more important for performance in men than in women, although we should not forget the significance that this vitamin may also have for women in other aspects, such as injury prevention [67].

In the triple hop test, once again there were no differences in performance between the sexes, and only the girls showed a negative correlation with vitamin D when the test was performed with the right leg, a result that was reinforced when BMI was also factored in. New gender differences in the association between vitamin D and performance seem to indicate a different use of strength in women and men in terms of the jumping actions involved in basketball performance. Notably, the lack of control of the menstrual cycle could have influenced our results. The majority of female subjects do not menstruate on a regular basis [68], and this factor was not considered at the time of blood sampling; however, despite the high coefficient of variation, our data did not show differences in iron concentration between male and female participants.

This study has several limitations. Firstly, the cross-sectional design of this study does not allow us to determine a direct cause and effect relationship. Comprehensive nutritional monitoring would have improved our knowledge of the origin of the vitamin D deficiencies found. Similarly, it is necessary to test whether vitamin D levels vary throughout the season and whether this is associated with a change in jump values in different periods. New and less invasive assessment systems based on tear biosensing or salivary samples [69,70] could streamline the process to obtain biomarkers during the competitive season, and therefore would allow relationships between strength and vitamin D to be analyzed from a more holistic (and rapid) view. Multidisciplinary teams—included nutritionists—regardless of the level of the sport club (elite and amateur), would facilitate the interpretation of these assessments, periodizated and tailored on a regular basis, therefore promoting health and young athletic success. Secondly, a larger sample size would allow more robust correlation coefficients to be obtained, for which reason our results cannot be extrapolated to other contexts and further studies are required. Finally, the differences found between men and women suggest that future studies should analyze whether the menstrual cycle somehow affects vitamin D, and thus sports performance in female basketball players, given their high incidence of injuries [71]. Some studies have demonstrated relationships between low levels of this vitamin and the frequency of menstrual disorders [72], confirming that this is a variable to control in these stages of development.

## 5. Conclusions

Our results suggest that, despite their youth, trainee basketball players have insufficient vitamin D levels. Since this deficiency appears to be common in elite athletes, especially those competing indoors, various means of controlling vitamin D levels throughout the season (diet, supplementation, and sun exposure) should be considered. Furthermore, these deficiencies appear to be differentially associated with jumping performance in men and women. Thus, while performance in men does seem to be compromised by low levels of this vitamin, it would be interesting to further investigate the different role it might play in women, as vitamin D deficiency is not only related to rate of force development.

## Figures and Tables

**Table 1:** Blood composition variables.

Parameters	Girls (*N* = 14)		Boys (*N* = 13)		*p*
	Mean ± *SD*	CV (%)	Mean ± *SD*	CV (%)	
Vitamin D (ng/mL)	29.14 ± 6.08	20.86	28.92 ± 6.40	22.13	0.905
Folic acid (ng/mL)	6.53 ± 3.38	51.76	7.24 ± 2.79	38.54	0.302
Cortisol (μg/dL)	15.37 ± 3.41	22.19	15.04 ± 1.86	12.37	0.616
Magnesium (mg/dL)	1.99 ± 0.60	30.15	2.08 ± 0.11	5.29	0.088
Iron (μg/dL)	87.35 ± 31.68	36.26	96.84 ± 38.72	39.98	0.491
Vitamin B12 (pg/mL)	559.78 ± 190.02	33.94	593.00 ± 177.33	29.90	0.643
TSH (μUI/mL)	2.74 ± 1.34	48.90	2.43 ± 0.87	35.80	0.491
Calcium (mg/dL)	9.59 ± 0.23	2.40	9.73 ± 0.21	2.16	0.105

CV: coefficient of variation in %; *SD*: standard deviation; TSH: serum thyroid stimulating hormone.

**Table 2:** Performance variables.

Tests	Girls (*N* = 14)		Boys (*N* = 13)		*p*
	Mean ± *SD*	CV (%)	Mean ± *SD*	CV (%)	
AbB (cm)	33.37 ± 4.83	14.47	35.71 ± 3.92	10.98	0.182
AbL (cm)	19.14 ± 4.32	22.57	24.14 ± 2.24 ^a,b^	9.28	0.005
AbR (cm)	20.31 ± 3.42	16.84	21.29 ± 2.99	14.04	0.436
THL (cm)	5.10 ± 0.70	13.72	6.10 ± 0.37	6.07	<0.001
THR (cm)	5.23 ± 0.69	13.19	6.13 ± 0.61	9.95	0.001

CV: coefficient of variation in %; *SD*: standard deviation; AbB: Abalakov bilateral; AbL: Abalakov left; AbR: Abalakov right; THL: triple hop left; THR: triple hop right. ^a^: Difference with the AbR of boys (*p* = 0.002); ^b^: Difference with the AbR of girls (*p* = 0.002).

**Table 3:** Correlations between jumping and hopping and vitamin D, considering both the whole sample, and male and female athletes separately, with and without the covariate body mass index (BMI).

Tests	Girls (*N* = 14)	Boys (*N* = 13)	All (*N* = 27)	Girls ^a^ (*N* = 14)	Boys ^a^ (*N* = 13)	All ^a^ (*N* = 27)
AbB (cm)	−0.611 *	0.703 **	0.047	−0.597 *	0.796 **	0.081
AbL (cm)	−0.219	0.218	−0.036	−0.183	0.248	−0.025
AbR (cm)	−0.465	−0.067	−0.227	−0.439	−0.040	−0.192
THL (cm)	−0.415	0.050	−0.106	−0.413	0.162	−0.098
THR (cm)	−0.666 **	0.128	−0.216	−0.685 **	0.210	−0.248

AbB: Abalakov bilateral; AbL: Abalakov left; AbR: Abalakov right; THL: triple hop left; THR: triple hop right; *: *p* < 0.05; **: *p* <0.01; ^a^: BMI as a covariate.

## Data Availability

Not applicable.
